# Evaporation of droplet in mid-air: Pure and binary droplets in single-axis acoustic levitator

**DOI:** 10.1371/journal.pone.0212074

**Published:** 2019-02-27

**Authors:** Yuki Niimura, Koji Hasegawa

**Affiliations:** Department of Mechanical Engineering, Kogakuin University, Hachioji, Tokyo, Japan; North China Electric Power University, CHINA

## Abstract

Acoustic levitation method (ALM) is a container-less processing method with applications in various fields, including material processing, biology, and analytical chemistry. Because it is a container-less processing technique, ALM could prevent nucleation and contamination of materials being processed via contact with a container wall. It is well-known that evaporation of a sample is an important process in container-less processing of materials; however, the mechanism of evaporation in multicomponent droplets in a single acoustic levitator is still unclear. Thus, we evaluate and understand the evaporation of an acoustically levitated multicomponent droplet and manipulate the evaporation process of the sample in this study. Specifically, we investigate the evaporation process of pure and multicomponent droplets using container-less processing experimentally. The evaporation processes and temporal evolution of the surface temperature of a multicomponent droplet were evaluated using a high-speed camera and radiation thermometer, respectively. We used water, ethanol, methanol, hexane, acetone, pentane, and binary solutions (solution of 25 wt%, 50 wt%, and 75 wt% ethanol, methanol, and acetone, respectively) as test samples to study the effect of saturated vapor pressure on evaporation. Ethanol, methanol, and acetone droplets evaporate in two different stages. It was observed that the water vapor in the air condensed during the evaporation process of these water-soluble droplets; hence, our experimental data did not agree with the theoretical prediction in accordance with the *d*^*2*^ law. Nevertheless, the evaporation behavior in the first stage of evaporation was consistent with the theoretical prediction. Furthermore, for binary droplets, as the concentration of the resultant solution increased owing to evaporation, the transition time from the first to the second stage of evaporation also increased. Based on these observations, estimation equations for binary droplets were developed to ensure that the experimental and theoretical values were in good agreement.

## Introduction

Acoustic levitation method (ALM) involves holding droplets without the need for a container using acoustic standing waves formed between a horn and reflector [1,2]. The container-less nature of ALM makes this technique widely applicable in the fields of material processing, biology, and analytical chemistry, since such container-less processing can prevent nucleation and contamination due to contact with container walls [2–9]. For example, Zang et al. applied ALM to the formation of bubbles for use in food, cosmetics, pharmaceuticals, ultra-light materials, and mineral flotation [10,11], additionally examining the spreading of potassium permanganate on the surface of levitated water droplets to develop a microreactor [12,13]. Xie et al. demonstrated that small organisms, such as insects and small fish can be acoustically levitated as well [14]. Furthermore, Sundvik et al. investigated the effect of levitating zebrafish embryo during hatching and growth processes [15]; their results indicated that there are no adverse effects of using levitation for transporting and observing organisms without contact, making levitation a useful tool for biological analysis. Vasileiou et al. demonstrated DNA transfection by transporting and mixing DNA of living organisms using the ALM [16]. In addition, Bouyer et al. introduced a technique to assemble three-dimensional (3D) cells in multiple layers using the ALM [17]. Furthermore, in recent years, several noncontact manipulation methods based on ultrasonic phased arrays have also been proposed [18–20].

As indicated above, though perfect sample manipulation using ALM has become important in recent times, this has not been achieved partly because of thermofluid nonlinearity. The nonlinear and dynamic behavior of an acoustically levitated droplet might affect its heat transfer, mass transport, and solidification properties [21–26], especially during the evaporation of a sample, which is an important process for container-less processing of materials. Yarin et al. theoretically studied the formulation of streaming flow around an acoustically levitated droplet; their results suggested that internal circulation might be caused by gas flow near the drop surface [27]. Furthermore, Hasegawa et al. studied the interaction between the evaporation behavior of levitated droplets and their internal as well as external flow structures [4]. Kobayashi et al. showed that there is a correlation between the internal as well as external flow structures in a levitated droplet and vapor concentration [28]. In addition, Bänsch et al. studied the temperature, vapor concentration, and flow structure of levitated droplets via numerical simulation [29]. Yarin et al. also developed a theoretical model of an acoustically driven droplet obtained via evaporation of binary mixtures [30]. Other theoretical models of evaporation for multicomponent droplets have been proposed [31–33].

Despite these past investigations exploiting the potential of the ALM, the mechanism of evaporation of multicomponent droplets in a single acoustic levitator is still unclear. In particular, it is important to understand the nonlinear behavior of the evaporation phenomenon on levitated multicomponent droplets. Thus, the objective of our study is to understand the evaporation mechanism of an acoustically levitated multicomponent droplet. In order to do so, we performed an experimental investigation on the evaporation process of pure and multicomponent droplets and compared the obtained results with those obtained using the existing *d*^*2*^ law.

## Materials and methods

### Experiment setup

**Fig 1:**
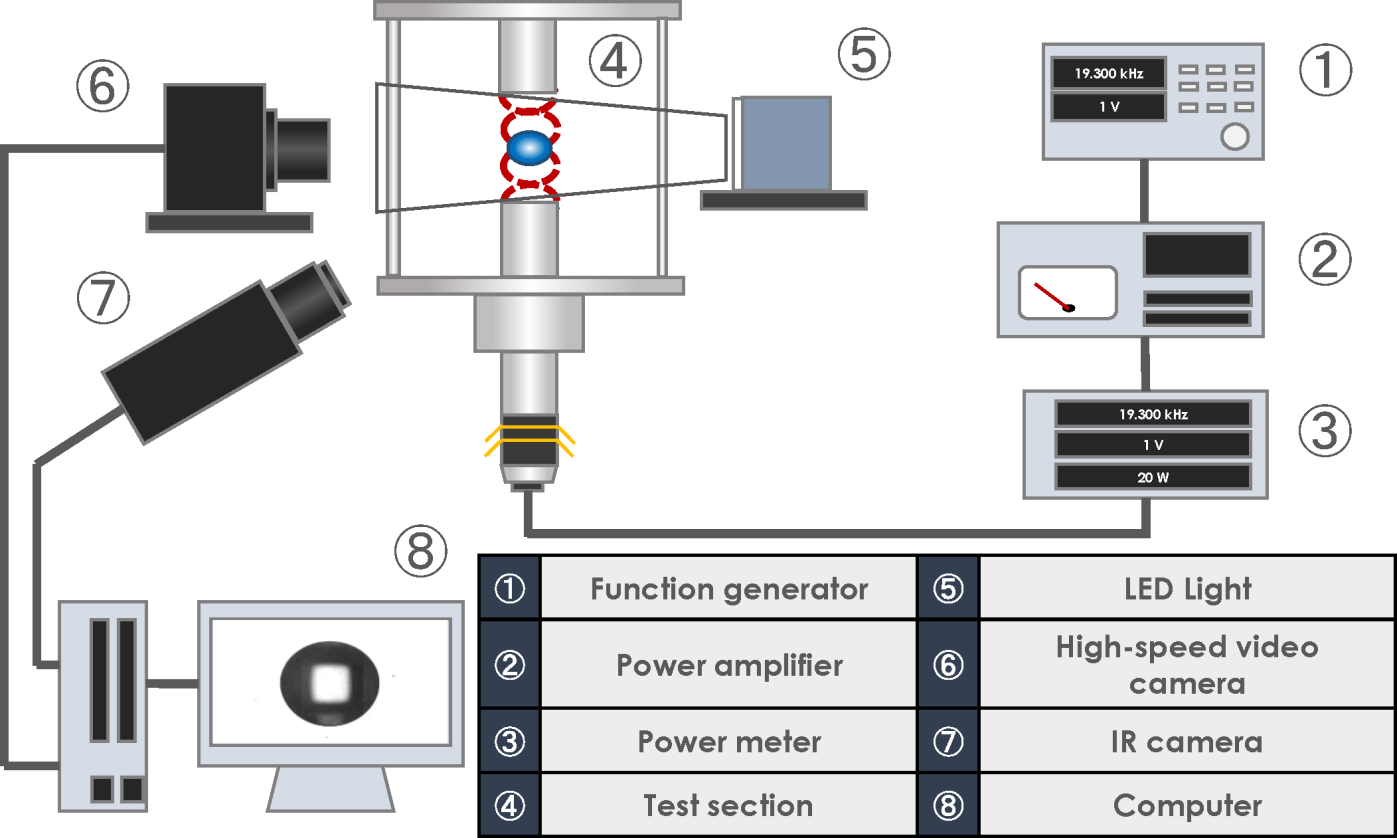
Schematic diagram of the experimental apparatus.

ALM allows one to truly suspend samples in mid-air, avoiding the solid-liquid interactions observed for conventional pendant drop and sessile droplet techniques. Consequently, it is crucial to observe the complete free surface of droplets and reveal the corresponding evaporation dynamics. Fig 1 shows a schematic diagram of the experimental apparatus used in this study. First, a sinusoidal signal is generated using a function generator (Agilent Technologies Japan, 33511B), after which this signal is amplified using a power amplifier (NF CORPORATION, 4502). Then, the amplified signal is inputted to an ultrasonic transducer (NGK SPARK PLUG CO., D4520PC) through a power meter (Yokogawa Test & Measurement Corporation, WT310-D-C1). Consequently, a sound wave is generated from the horn placed at the bottom, which is then reflected by the top reflector, leading to the formation of an acoustic standing wave between the horn and reflector. The droplet is manually injected near a pressure node of the acoustic standing wave using a syringe. This droplet can be levitated near a pressure node of the acoustic standing wave. To visualize the behavior of a levitated droplet, we capture the levitated droplet under backlight illumination using a high-speed camera (PHOTRON, FASTCAM Mini AX50); in addition, the temporal evolution of the surface temperature of the droplet was recorded using a radiation thermometer (FLIR Systems, A6750sc MWIR). Finally, the obtained images were processed using the MATLAB Image Processing Toolbox [34] to quantify droplet diameter.

Table 1 lists the experimental conditions used in our study. In particular, the function generator had an operating frequency of approximately 19.3 kHz. The distance between the horn and reflector was 48 mm. The sound pressure in the test section was 1.2–1.9 kPa. We used water (Sanei Corporation), ethanol (KENEI PHARMACEUTICAL CO.), methanol, hexane, acetone, pentane (FUJIFILM Wako Pure Chemical Corporation), and a binary fluid (ethanol and methanol solutions) as test samples to evaluate the effect of saturated vapor pressure on the evaporation process. An initial diameter is defined as an equivalent diameter. An aspect ratio is the ratio of major diameter *b* to minor diameter *a*.

**Table 1:** Experimental conditions.

Input frequency *f*	19.3 kHz
Horn-reflector gap	48 mm
Sound pressure *P*_*rms*_	1.0–1.9 kPa
Temperature *T*	25±2°C
Relative Humidity *RH*	25%, 50%±10%
Test sample	Water, ethanol, methanol, hexane, acetone, pentane, ethanol solution, methanol solution
Wave length	17.9 mm
Initial diameter *d*_*0*_	0.9–1.7 mm
Aspect ratio *b/a*	1.1–1.8

### Statistical analysis

The levitated droplet was captured using a high-speed camera with a spatial resolution of ~20 μm/pixel to ensure that the measurement error associated with the droplet diameter is below 3%. In addition, the sound pressure was measured thrice, and the error was restricted to a maximum of 5%. Finally, the surface temperature of the droplet measured using the radiation thermometer included a ± 2°C error.

## Results and discussion

### Evaporation process of the levitated pure droplet

**Fig 2:**
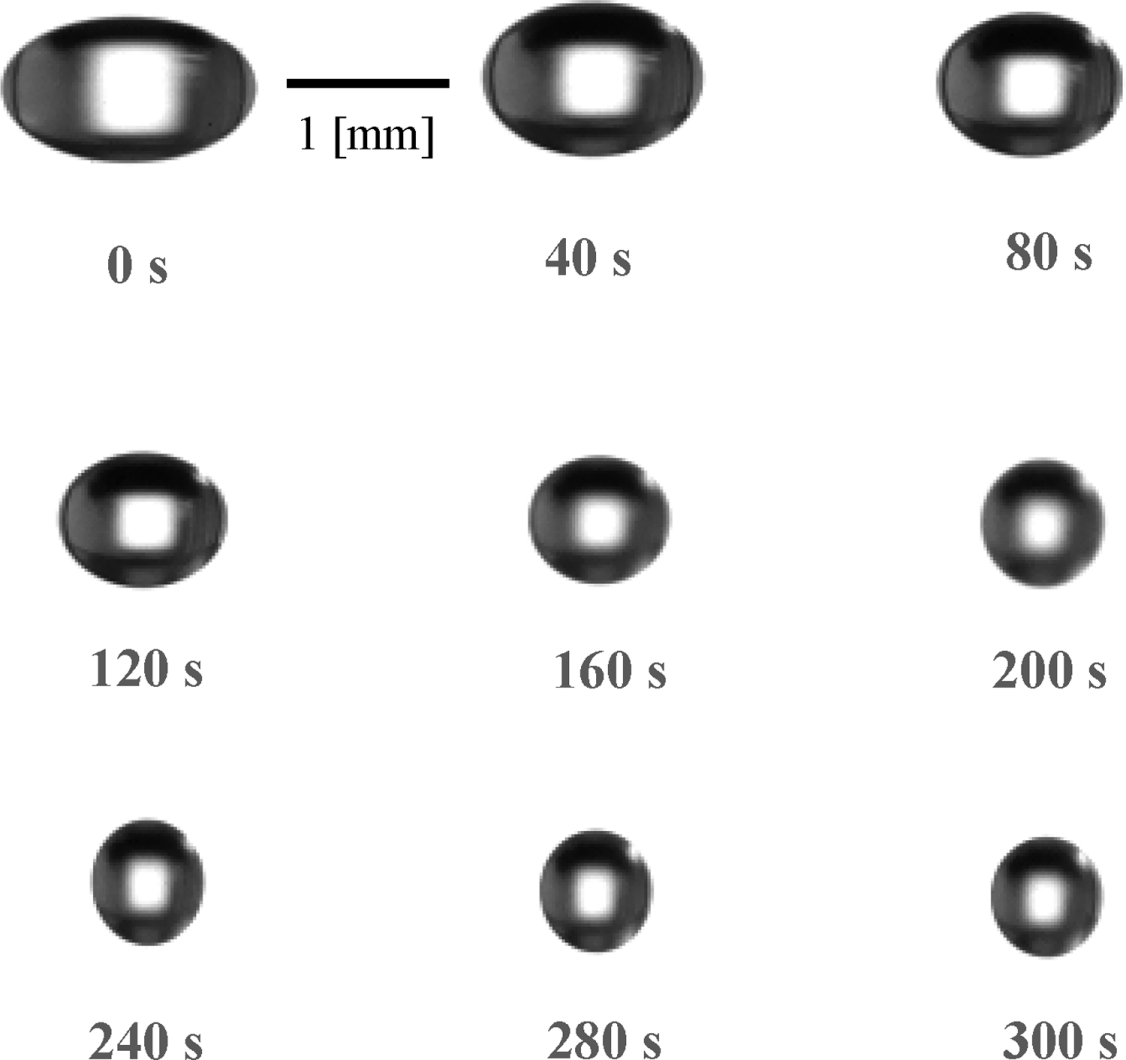
Evaporation process of the ethanol droplet.

**Fig 3:**
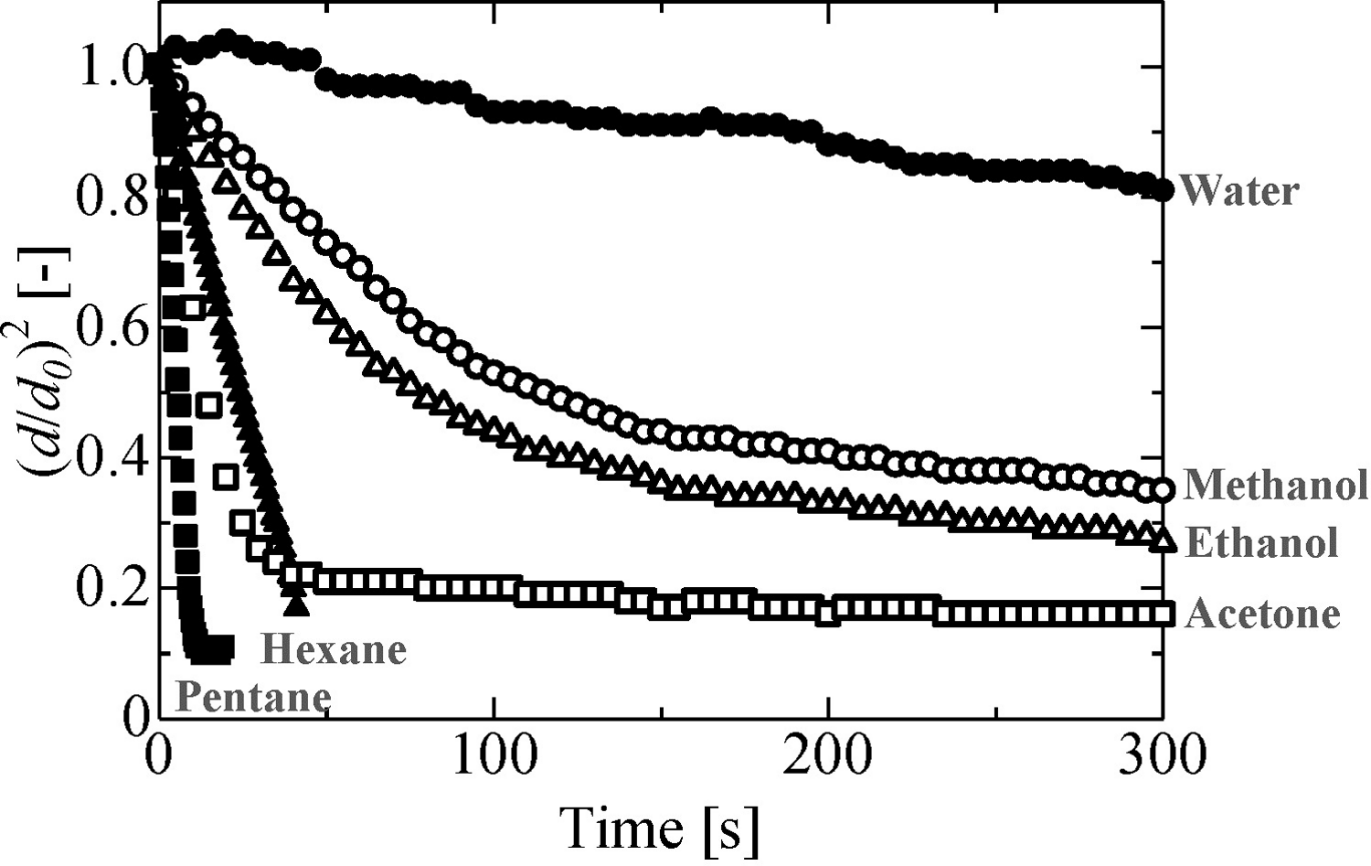
Evaporation process of the levitated pure droplet. Water: *d*_*0*_ = 1.4 mm, *P*_*rms*_ = 1.8 kPa, *b/a* = 1.2, *P*_*s*_ = 3534 Pa, Ethanol: *d*_*0*_ = 1.4 mm, *P*_*rms*_ = 1.5 kPa, *b/a* = 1.7, *P*_*s*_ = 8850 Pa, Methanol: *d*_*0*_ = 1.4 mm, *P*_*rms*_ = 1.5 kPa, *b/a* = 1.6, *P*_*s*_ = 15817 Pa, Hexane: *d*_*0*_ = 1.6 mm, *P*_*rms*_ = 1.0 kPa, *b/a* = 1.3, *P*_*s*_ = 20125 Pa, Acetone: *d*_*0*_ = 1.2 mm, *P*_*rms*_ = 1.4 kPa, *b/a* = 1.6, *P*_*s*_ = 28061 Pa, Pentane: *d*_*0*_ = 0.9 mm, *P*_*rms*_ = 1.0 kPa, *b/a* = 1.1, *P*_*s*_ = 67973 Pa.

Fig 2 depicts the evaporation process of an ethanol droplet. It was confirmed from our obtained images that the levitated droplet area decreased with time. In the ALM, levitation is achieved by the application of sound pressure above and below the droplet. Although surface tension forces try to preserve the spherical shape of the droplet, levitating droplets exhibit an ellipsoidal shape due to the effect of sound pressure. As the droplet diameter decreases with time, surface tension becomes progressively more dominant, and the droplet regains its spherical shape. Fig 3 depicts the evaporation process of a levitated single-component droplet; in this figure, the horizontal axis indicates the square of droplet diameter *d* normalized by the square of initial droplet diameter *d*_*0*_. As can be seen from Fig 3, the area of the droplet surface decreased with time in all cases. In particular, in the cases of water, hexane, and pentane droplets, the surface area of the droplet linearly decreased during the evaporation process; in contrast, in the cases with ethanol, methanol, and acetone droplets, the observed evaporation behavior was different—these droplets evaporated in two different stages. These stages can be attributed to the condensation of water in the air over the droplets of ethanol, methanol, and acetone during the evaporation process, consequently affecting the evaporation process of the soluble material itself.

**Fig 4:**
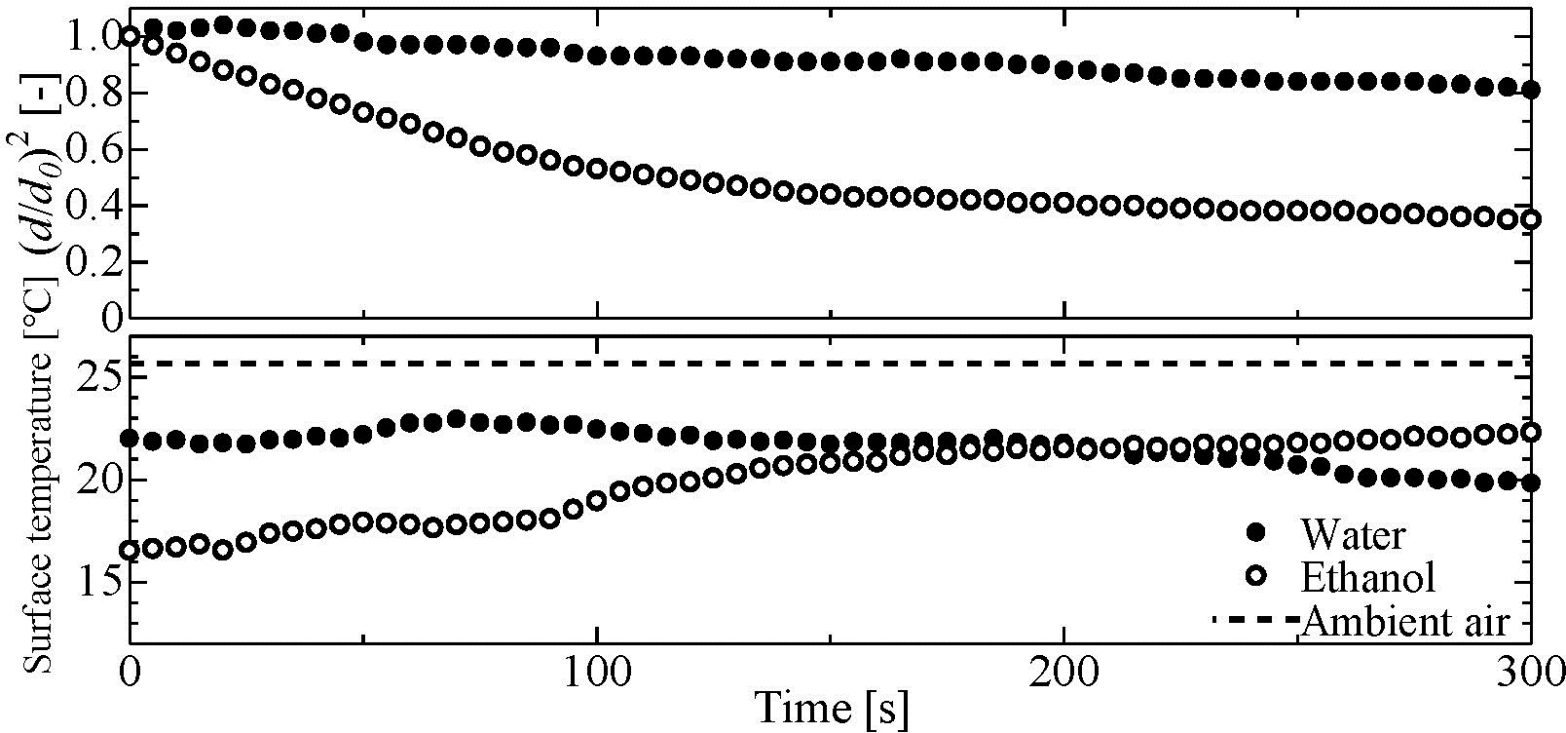
Surface temperature of water and ethanol droplets as a function of time.

Fig 4 shows the surface temperature of water and ethanol droplets, with top and bottom graphs presenting the evaporation process and surface temperature of ethanol droplets, respectively. In particular, the surface temperatures of water and ethanol droplets were lower than the ambient air temperature, which can be attributed to the latent heat of vaporization. Furthermore, while the surface temperature of water droplets was constant during their evaporation, the surface temperature of ethanol droplets rose during their evaporation. However, the surface temperature of ethanol droplets was about the same as that of water droplets at 140 s. This is because, owing to evaporation and condensation, leaving only water droplets at 140 s.

The heat loss of a given droplet, described by droplet evaporation–induced latent heat and mass loss, strongly influences the evaporation process by changing droplet surface temperature. We estimated the thermal balance between droplet evaporation and heat transfer around a droplet as follows.

$$hA\left(T_{s}-T_{\infty }\right)=\rho_{l}L\frac{dV}{dt}$$ (1)

Ts−T∞=ρlLdVdthA (2)

where *T*_*s*_ is the droplet surface temperature, *T*_*∞*_ is the room temperature, $\rho_{l}$ is the density of the liquid, *V* (= π*d*^3^/6) is the droplet volume, *t* is time, *h* is the heat transfer coefficient, and *A* (= π*d*^2^) is the droplet surface area. The change of droplet volume with time (*dV/dt*) was estimated up to a time of 50 s by linear approximation, and the value of the heat transfer coefficient was taken from our previous study [24]. Substitution of ρ = 785 kg/m^3^, *L* = 838 kJ/kg, *dV/dt* = 1.15 × 10^−11^ m^3^/s, *h* ~ 100 W/(m^2^ K), and *A* = 6.47 × 10^−6^ m^2^ into Eq 2 allows the temperature difference $T_{s}-T_{\infty }$for an ethanol droplet to be calculated as ~10°C, which is in good agreement with the initial temperature drop (~10°C) of ethanol shown in Fig 4. For a water droplet, the above temperature was calculated as ~ 5°C, also in good agreement with the results presented in Fig 4.

**Fig 5:**
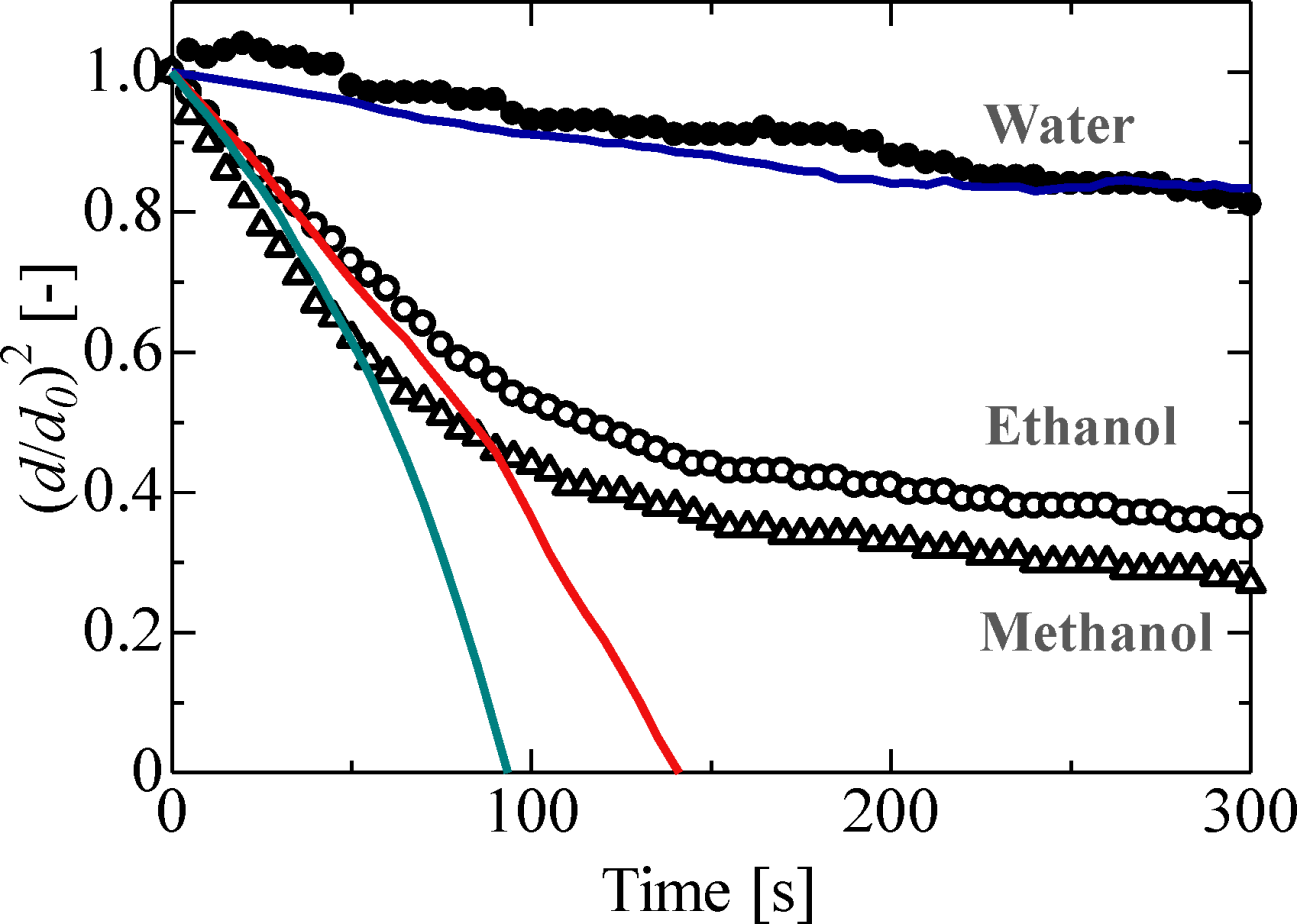
Comparison of theoretical and experimental (*d*/*d*_0_)^2^ value evolution with time. Theoretical values calculated using the *d*^*2*^ law are indicated by blue (water) red (ethanol), and green (methanol) solid line.

Fig 5 shows a comparison of the theoretical and observed results for the evaporation of water and ethanol droplets. Evaporation processes of a levitated droplet in a gaseous environment can be described using (Eq 3) [35] as follows:

$${\left(\frac{d}{d_{0}}\right)}^{2}=1-\frac{8DM}{\rho_{l}R}\left(\frac{P_{s}}{T_{s}}-\frac{P_{\infty }}{T_{\infty }}\right)\frac{t}{d_{0}^{2}}$$ (3)

where *D* is the diffusion coefficient, *M* is the molecular weight, *R* is the gas constant, *P*_*s*_ is the vapor pressure at droplet surface, and *P*_*∞*_ is the vapor pressure in the air. The solid line in Fig 5 corresponds to the theoretical evaporation values derived using the *d*^*2*^ law. The theoretical and experimental evaporation results for water droplets are in good agreement with each other; however, those for ethanol and methanol droplets are not. Furthermore, the evaporation behavior in the first stage is consistent, but the same in the second stage cannot be concluded. This nonlinear decrease in droplet surface area in the case of ethanol droplets can be attributed to the condensation of surrounding water vapor.

**Fig 6:**
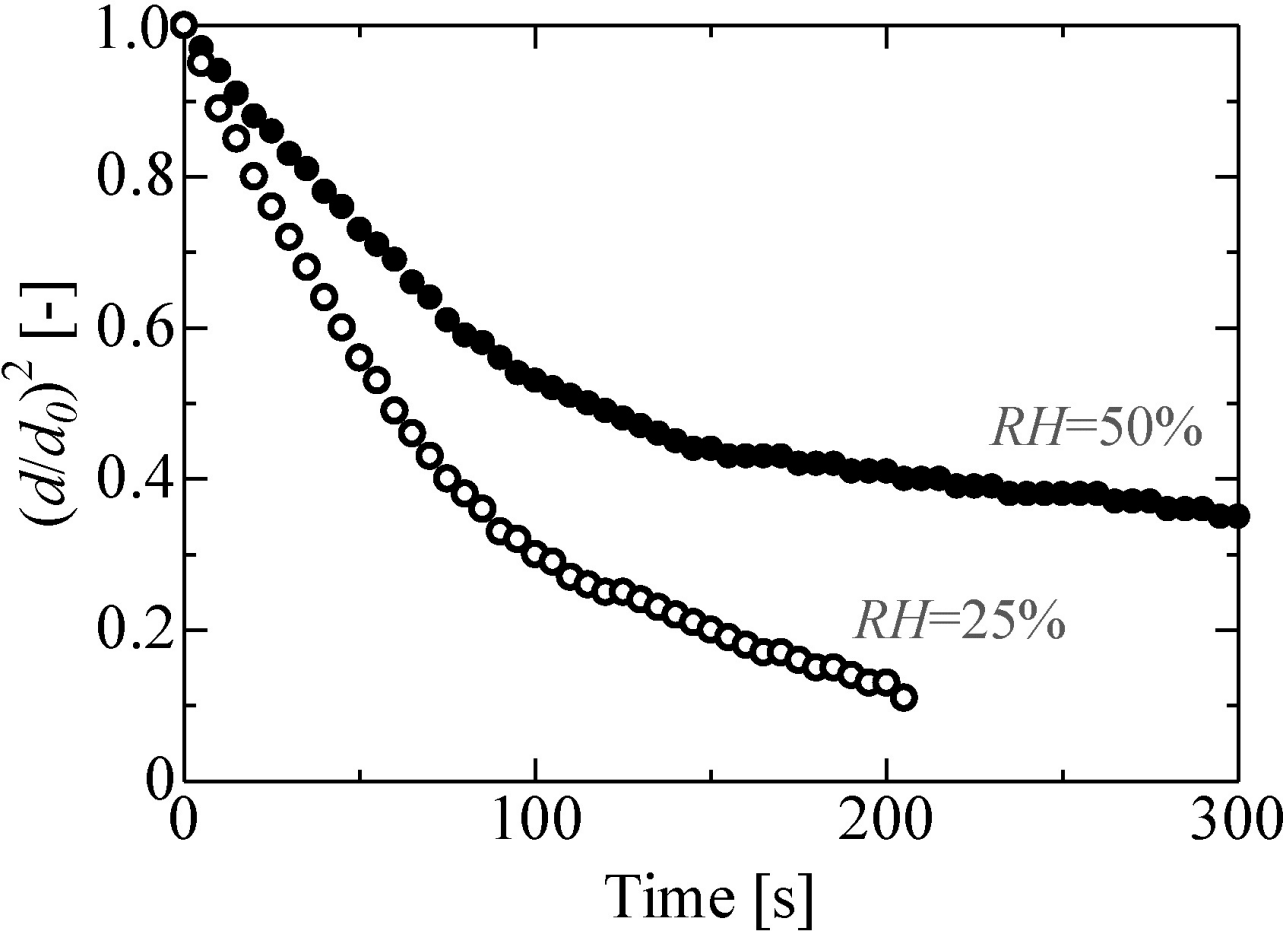
Effect of relative humidity on the evaporation process of an ethanol droplet. *RH* = 50%: *d*_*0*_ = 1.4 mm, *P*_*rms*_ = 1.5 kPa, *b/a* = 1.7, *RH* = 25%: *d*_*0*_ = 1.4 mm, *P*_*rms*_ = 1.3 kPa, *b/a* = 1.4.

Fig 6 shows the effects of relative humidity on the evaporation process of ethanol droplets, revealing the influence of ambient air condensation. In our study, we considered relative humidities of 25% and 50%. It was observed that the evaporation rate was higher at 25% relative humidity than that at 50% relative humidity. Furthermore, the evaporation process with a relative humidity of 50% was a two-stage process, whereas the evaporation process with a relative humidity of 25% showed a linear trend, which, as previously mentioned, can be attributed to the formation of an aqueous ethanol solution because of the condensation of surrounding water vapor in the air on the ethanol droplet.

### Evaporation process of levitated binary droplets

**Fig 7:**
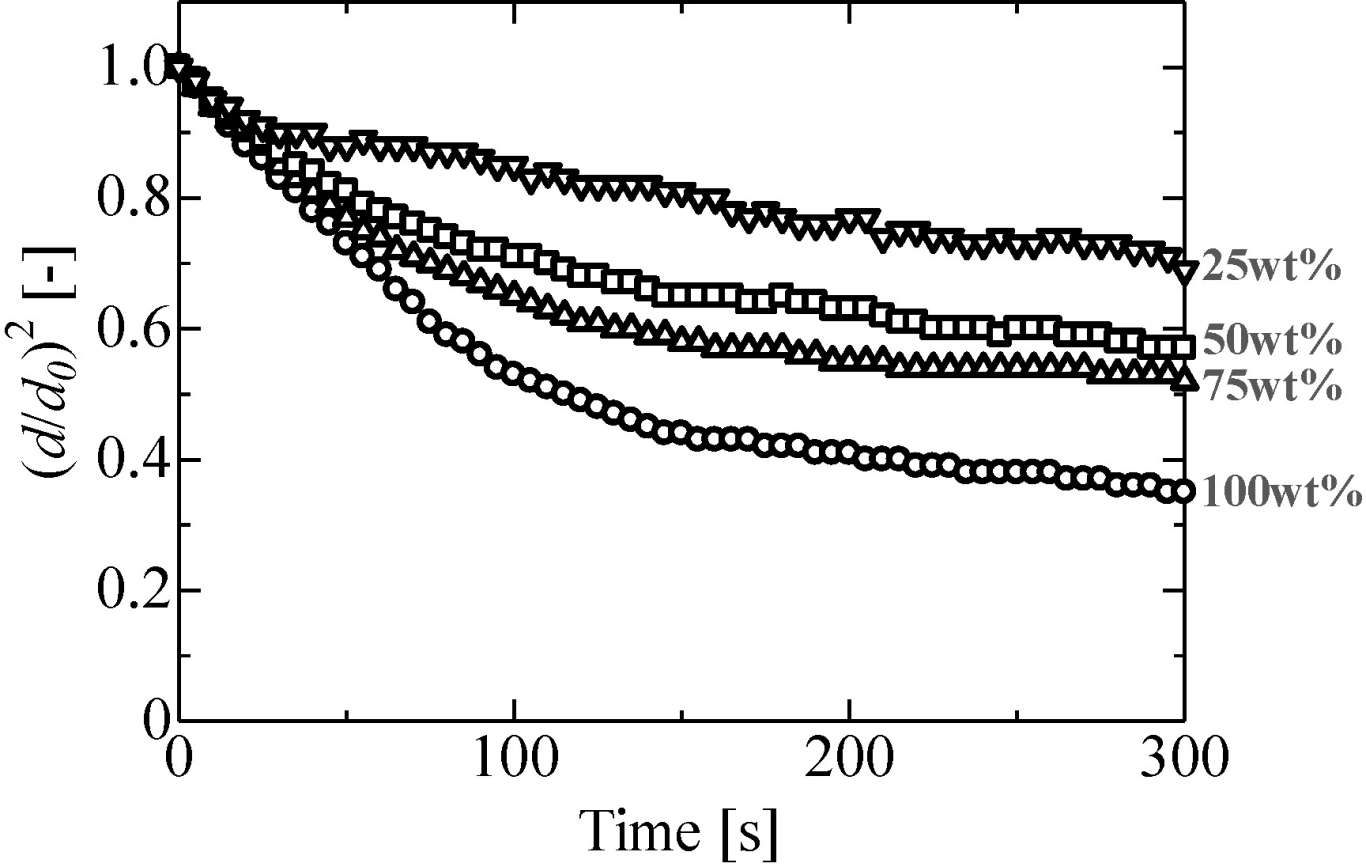
Evaporation process of ethanol solution droplets. 100 wt%: *d*_*0*_ = 1.4 mm, *P*_*rms*_ = 1.5 kPa, *b/a* = 1.7, 75 wt%: *d*_*0*_ = 1.4 mm, *P*_*rms*_ = 1.7 kPa, *b/a* = 1.8, 50 wt%: *d*_*0*_ = 1.5 mm, *P*_*rms*_ = 1.5 kPa, *b/a* = 1.5, 25 wt%: *d*_*0*_ = 1.4 mm, *P*_*rms*_ = 1.8 kPa, *b/a* = 1.3.

**Fig 8:**
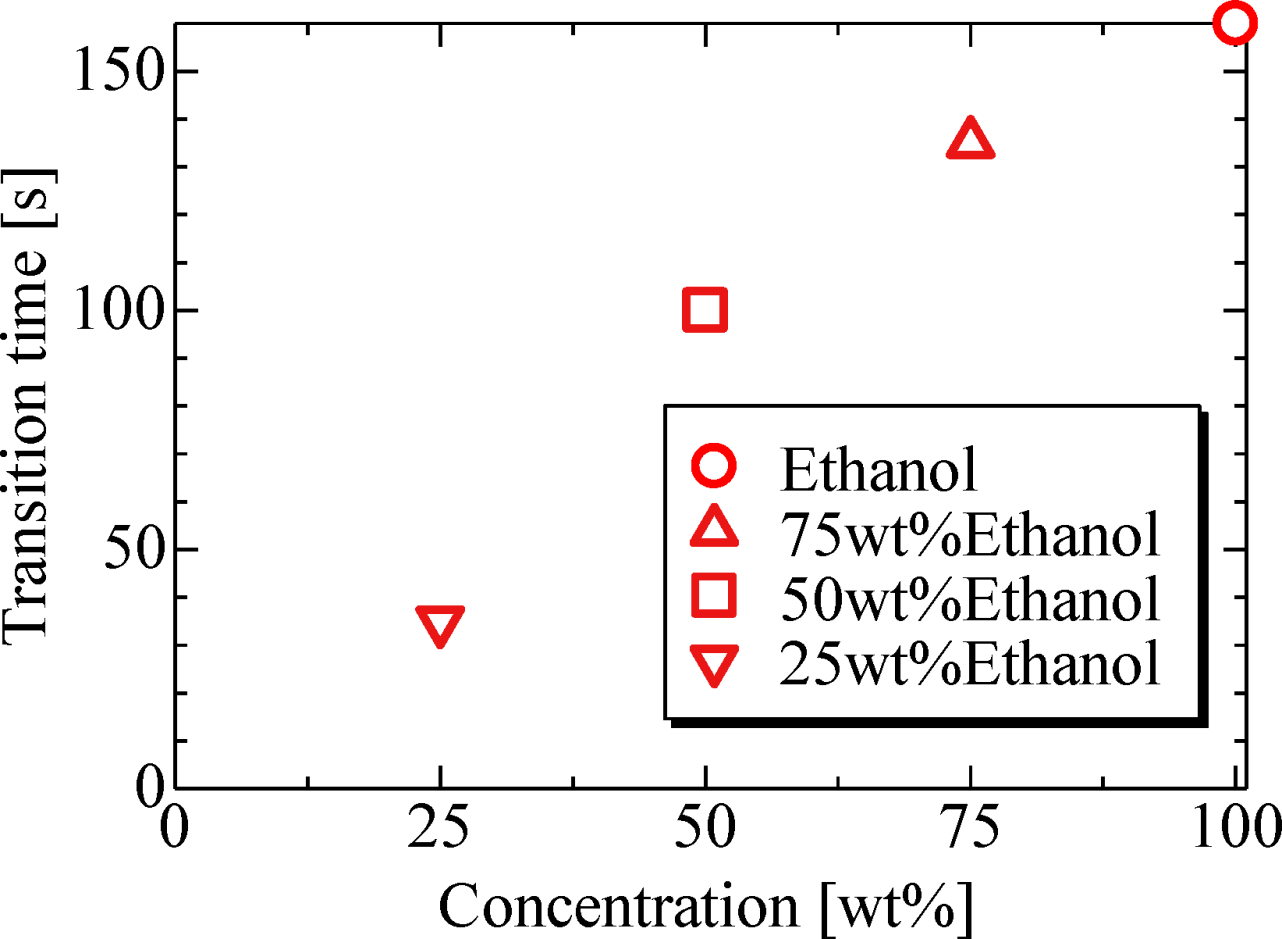
Transition time of ethanol solution droplets.

Fig 7 shows the evaporation process of ethanol solution droplets. Three ethanol concentrations of 25 wt%, 50 wt%, and 75 wt% were considered in our study. The ethanol solution droplets showed the same evaporation behavior as pure ethanol, i.e., the evaporation process of ethanol solution also involves two stages. Furthermore, as the concentration increased owing to evaporation, the transition time from the first to second stage also increased because of preferential evaporation. The transition time trend is shown in Fig 8. The experimental transition time is defined as the time when the evaporation rate of an originally binary droplet became of the same order of magnitude as that of a water droplet from Fig 7. However, when ethanol concentration eventually decreased, so did the transition time between stages.

The transition time can also be predicted using the *d*^*2*^ law given by (Eq 3). When the droplet diameter *d* = 0 [mm] in (Eq 3), it implies that the droplet has fully evaporated. For a binary droplet, *t*_*trans*_ represents the transition time at which only ethanol or methanol primarily evaporates and leaving only water, which can be obtained using (Eq 4) as follows:

$$t_{trans}=\frac{d_{0}^{2}}{\beta },\;\beta =\frac{8DM}{\rho_{l}R}\left(\frac{P_{s}}{T_{s}}-\frac{P_{\infty }}{T_{\infty }}\right)$$ (4)

**Fig 9:**
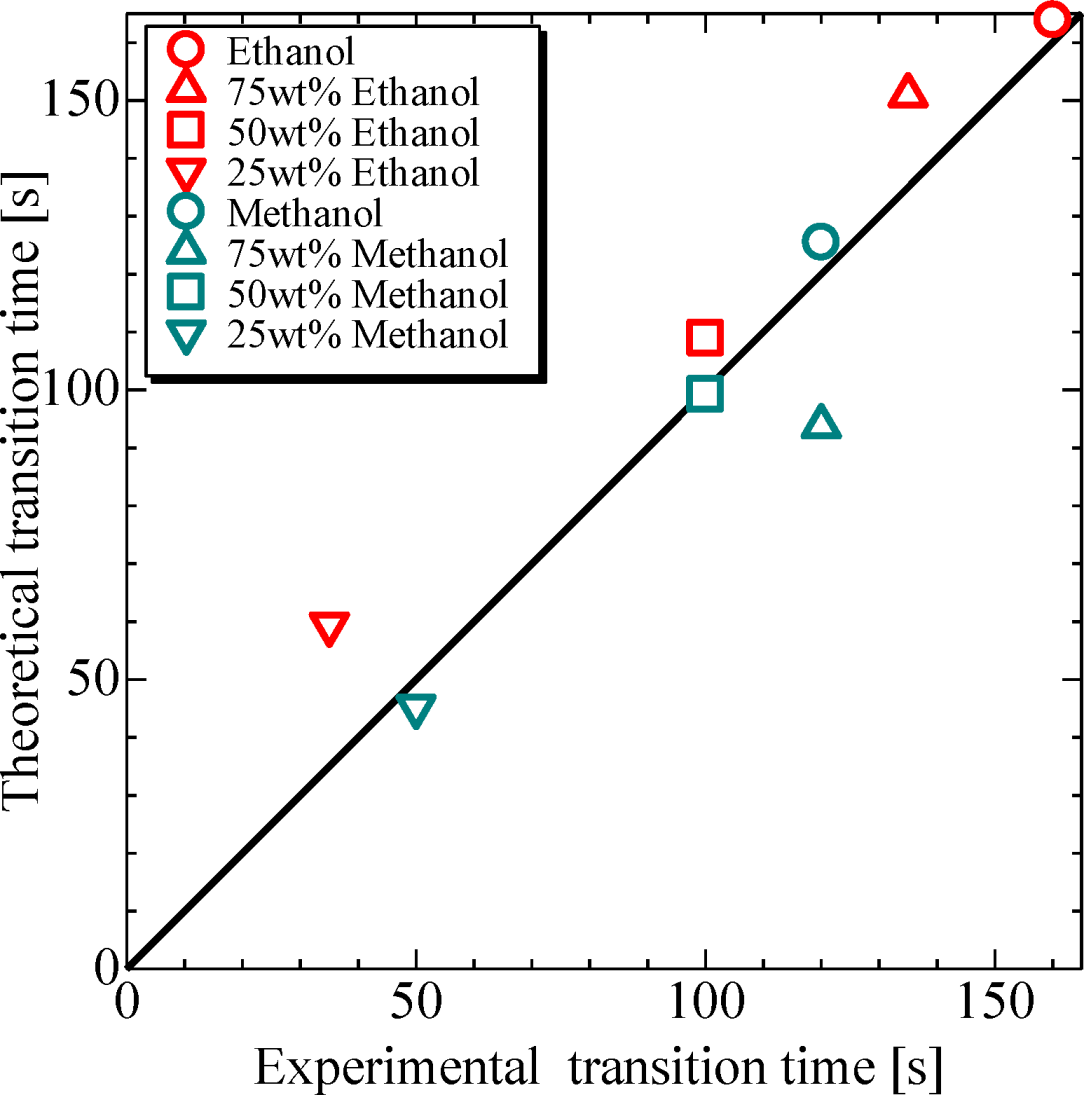
Experimental and theoretical transition time of ethanol and methanol solution droplets. (Ethanol) 100 wt%: *d*_*0*_ = 1.4 mm, *P*_*rms*_ = 1.5 kPa, *b/a* = 1.7, 75 wt%: *d*_*0*_ = 1.4 mm, *P*_*rms*_ = 1.7 kPa, *b/a* = 1.8, 50 wt%: *d*_*0*_ = 1.5 mm, *P*_*rms*_ = 1.5 kPa, *b/a* = 1.5, 25 wt%: *d*_*0*_ = 1.4 mm, *P*_*rms*_ = 1.8 kPa, *b/a* = 1.3. (Methanol) 100 wt%: *d*_*0*_ = 1.4 mm, *P*_*rms*_ = 1.5 kPa, *b/a* = 1.6, 75 wt%: *d*_*0*_ = 1.7 mm, *P*_*rms*_ = 1.5 kPa, *b/a* = 1.5, 50 wt%: *d*_*0*_ = 1.5 mm, *P*_*rms*_ = 1.5 kPa, *b/a* = 1.4, 25 wt%: *d*_*0*_ = 1.5 mm, *P*_*rms*_ = 1.9 kPa, *b/a* = 1.5.

Fig 9 shows the comparison of theoretically and experimentally obtained transition time values. As can be deduced from the results, the experimental and theoretical values are in good agreement, including those for premixed droplets, which indicates that the transition time of evaporation behavior can be predicted.

**Fig 10:**
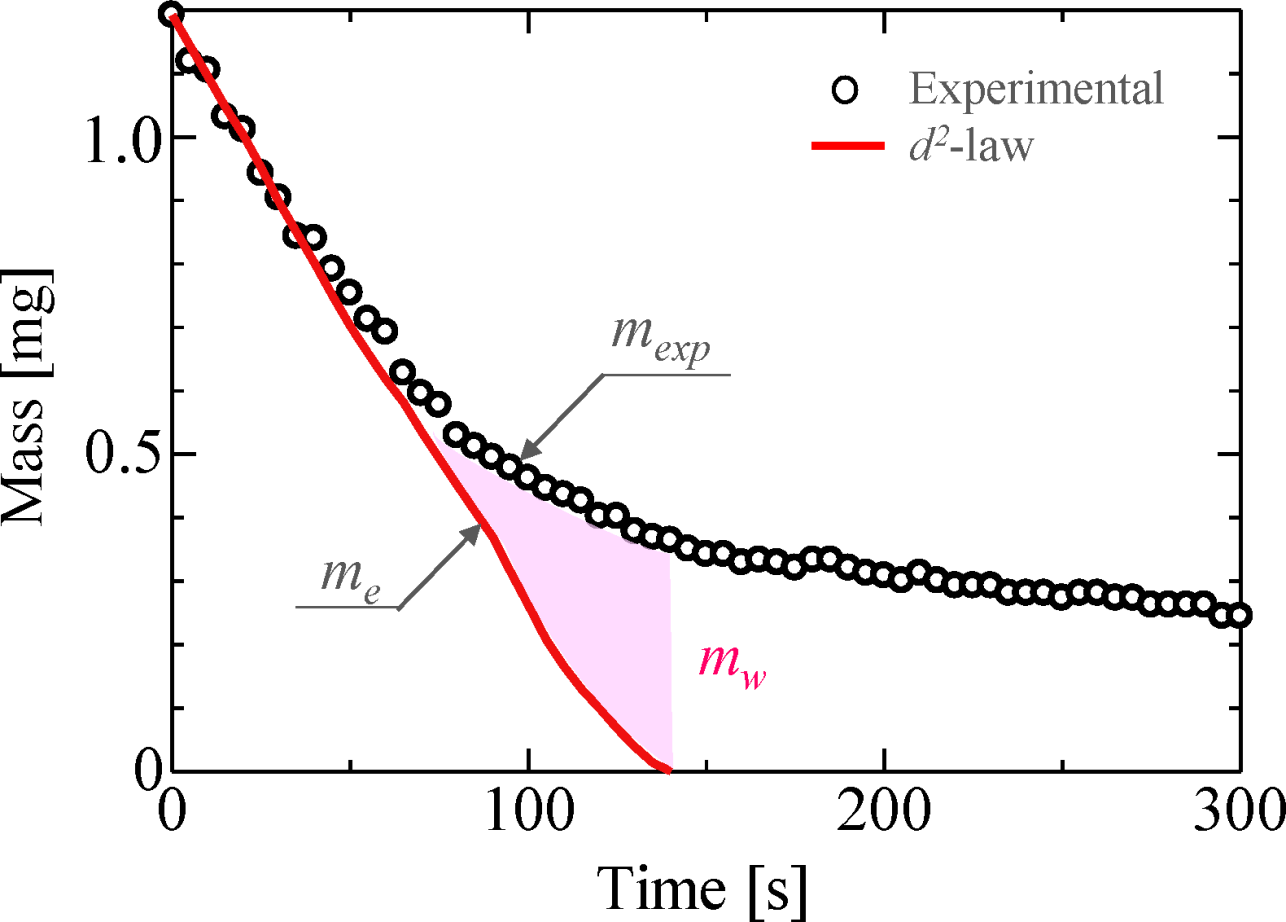
Conceptual diagram of mass estimation of ethanol droplets.

Based on the abovementioned transition time and concentration estimations, we developed an estimation equation for multicomponent droplets. First, the concentration estimation method was performed, which is as follows. In order to estimate the concentration, the mass of each component is calculated. The conceptual diagram of the mass estimation model for a binary droplet is shown in Fig 10; in particular, the plot in the figure shows the experimental value, while the solid line shows the theoretical values for the ethanol droplet. First, the mass of ethanol component in the droplet is calculated based on the equivalent diameter at each time point using (Eq 5). Furthermore, the mass of the water component in the droplet is expressed as the difference between the experimental and theoretical values using (Eq 6). The highlighted portion in the figure represents the mass of the water component.

$$m_{e}=\rho_{e}\frac{4}{3}\pi {\left(\frac{d_{th}}{2}\right)}^{3}$$ (5)

$$m_{w}=m_{exp}-m_{e}$$ (6)

**Fig 11:**
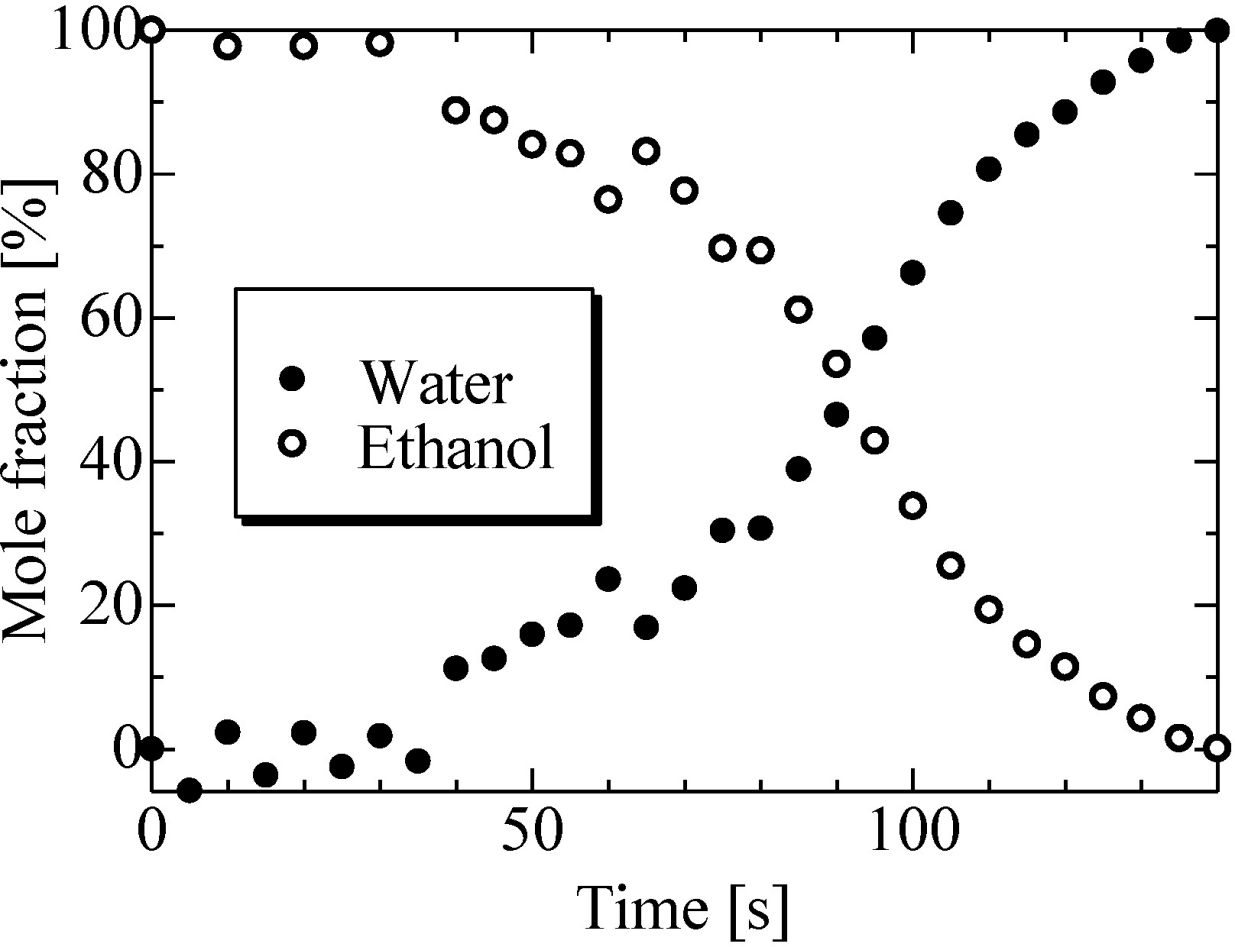
Evolution of ethanol droplet composition with time.

The mass fractions of ethanol and water were calculated using Eqs (7) and (8) based on the mass of each component obtained using Eqs (5) and (6), respectively. In addition, the mole fractions were calculated using Eqs (9) and (10) based on the mass fraction of each component. Fig 11 shows the temporal evolution of ethanol droplet composition, revealing that at 90 s, the concentration of ethanol became lower than the concentration of water and decreased further, eventually reaching zero at 140 s (i.e., the droplets contained only water after this time).

$$Y_{e}=\frac{m_{e}}{m_{e}+m_{w}}$$ (7)

$$Y_{w}=\frac{m_{w}}{m_{e}+m_{w}}$$ (8)

$$Z_{e}=\frac{Y_{e}M_{w}}{Y_{e}M_{w}+\left(1-Y_{e}\right)M_{e}}$$ (9)

$$Z_{w}=\frac{(1-Y_{e})M_{e}}{Y_{e}M_{w}+\left(1-Y_{e}\right)M_{e}}$$ (10)

The equations for modeling multicomponent droplets were constructed based on the estimated mole fractions at each time. The estimation equation is given by (Eq 11). In particular, the evaporation variables for two-component droplets are calculated by determining *β* for each component described by Eqs (12) and (13), multiplying the result with the mole fraction, and summing each value of *β* as shown in (Eq 14). For this estimation equation we assume Raoult's law to be valid.

$${\left(\frac{d}{d_{0}}\right)}^{2}=1-\beta_{mix}\frac{t}{d_{0}^{2}}$$ (11)

$$\beta_{e}=\frac{8D_{e}M_{e}}{\rho_{e}R}\left(\frac{Z_{e}P_{se}}{T_{s}}-\frac{P_{\infty }}{T_{\infty }}\right)$$ (12)

$$\beta_{w}=\frac{8D_{w}M_{w}}{\rho_{w}R}\left(\frac{Z_{w}P_{sw}}{T_{s}}-\frac{P_{\infty }}{T_{\infty }}\right)$$ (13)

$$\beta_{mix}=\beta_{e}+\beta_{w}$$ (14)

**Fig 12:**
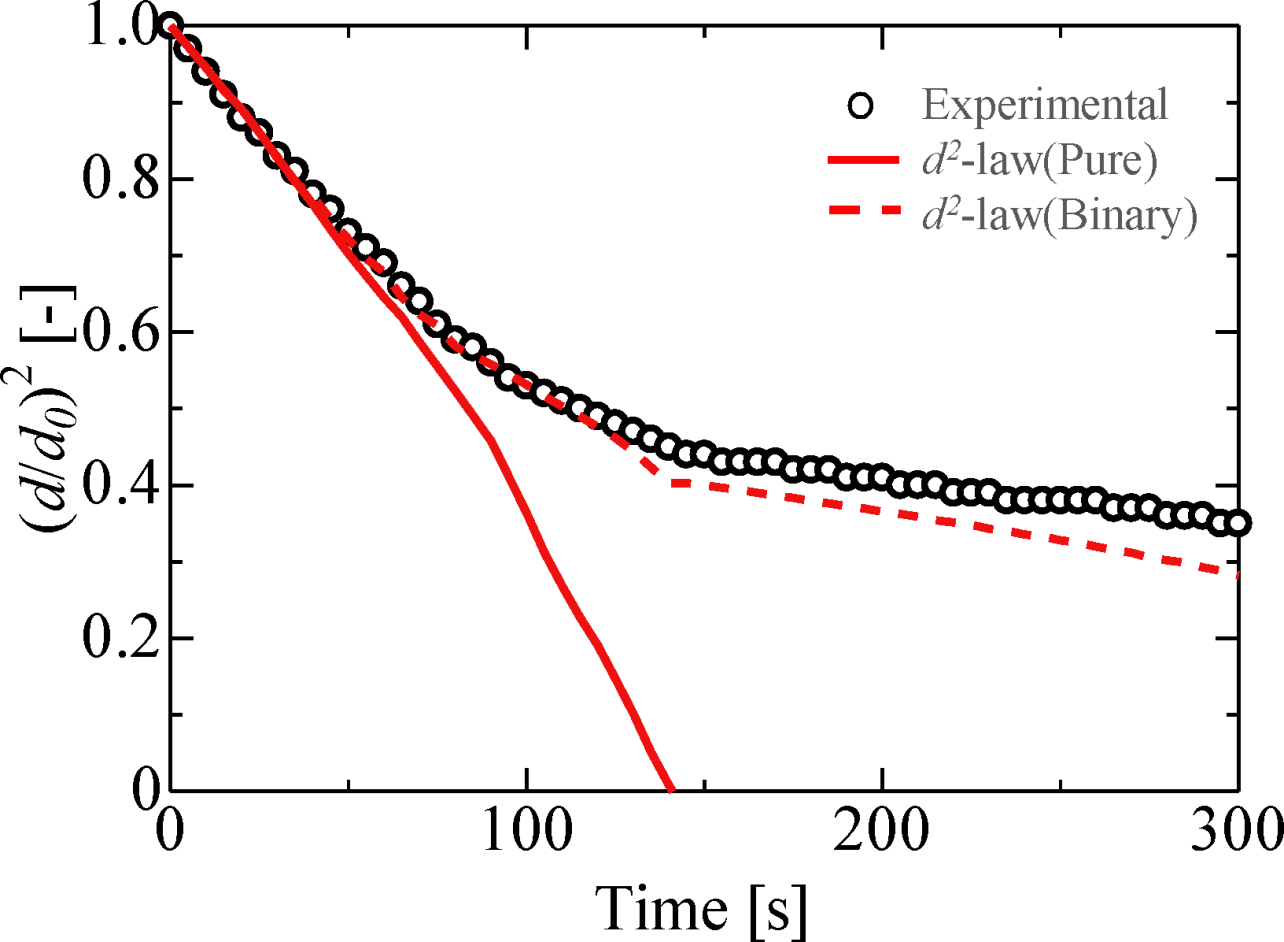
Re-evaluation of evaporation process for ethanol droplets. *d*_*0*_ = 1.4 mm, *P*_*rms*_ = 1.5 kPa, *b/a* = 1.7.

Fig 12 shows the results of the re-evaluated experimental values based on the proposed estimation equation. The dashed line in the figure represents the result obtained using the original estimation equation. It was observed that the experimental results agreed with those obtained using the estimation equation considering concentration change. Thus, it was confirmed that the evaporation behavior of multicomponent droplets under acoustic levitation conditions can be identified by the proposed model. These findings can be used to understand and predict evaporation processes in acoustically levitated droplets for potential lab-on-a-drop applications [36], including blood analysis, pharmaceutical, and food drying processes. In the future, we plan to investigate the direct measurement of flow and vapor concentration fields around a levitated droplet for contactless droplet manipulation.

## Conclusions

In this study, we experimentally investigated the evaporation process of pure and multicomponent droplets using acoustic levitation and compared our experimentally obtained results with theoretical results. Because ethanol, methanol, and acetone droplets are highly soluble in water, it is assumed that the surrounding water vapor condensed onto the droplets leading to two different evaporation stages. Furthermore, because of the effect of ambient humidity, the ethanol droplets showed a linear evaporation behavior, indicating that the surrounding water vapor condensed onto the ethanol droplets, gradually leading to the formation of an aqueous ethanol solution. However, as the concentration of the ethanol or methanol solution increased due to evaporation, the transition time from the first to second stage also increased due to preferential evaporation. Our results indicate that transition times between stages of evaporation behavior could be predicted, including for premixed droplets. Finally, we constructed an estimation equation that considers this change in concentration over time. Our results suggest that the evaporation behavior of multicomponent droplets under acoustic levitation could be predicted.
